# Supplementary material for: California’s zero-emission vehicle adoption brings air quality benefits yet equity gaps persist
Source: Nat Commun. 2023 Dec 12;14:7798. doi: 10.1038/s41467-023-43309-9 (PMC10716132; doi:10.1038/s41467-023-43309-9)
Supplement: Supplementary file 1 — Supplementary Information [file 41467_2023_43309_MOESM1_ESM.pdf]

Supplementary Information for

California's zero-emission vehicle adoption brings air quality benefits yet equity gaps persist

Qiao Yu[1], Brian Yueshuai He[2], Jiaqi Ma[2], Yifang Zhu[1*]

1. Department of Environmental Health Sciences, Fielding School of Public Health, University of California, Los Angeles, Los Angeles, CA, United States

2. Department of Civil and Environmental Engineering, Samueli School of Engineering, University of California, Los Angeles, Los Angeles, CA, United States

* Corresponding Author: Yifang Zhu; e-mail: yifang@ucla.edu

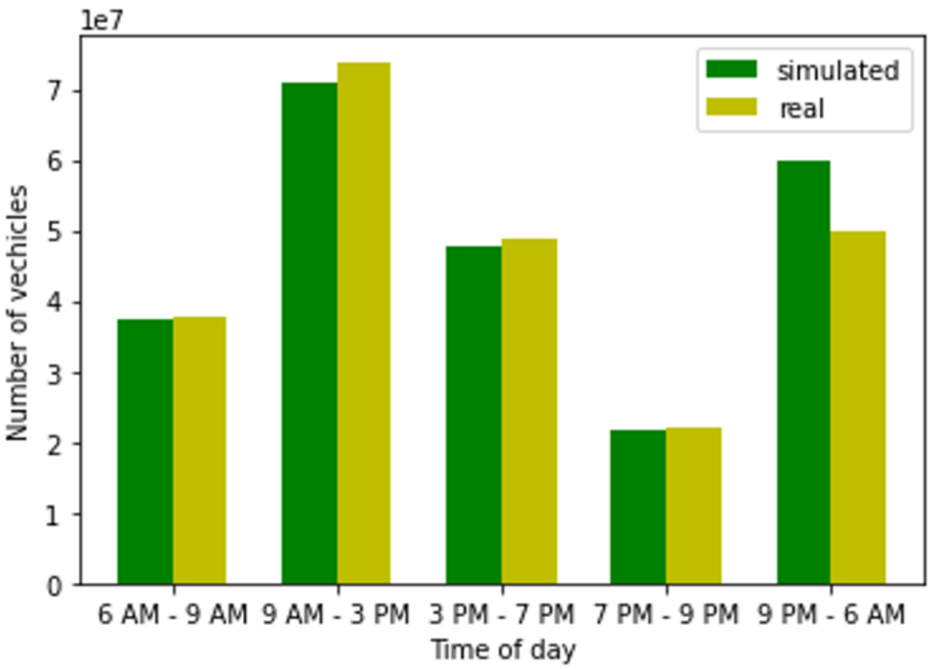

Figure S1. Comparison of simulated and real traffic volumes in Los Angeles County.

Table S1 | Zero-emission vehicle (ZEV) ownership, electric vehicle miles traveled (eVMT), and traffic-emitted air pollutants in disadvantaged communities (DACs) vs. non-DACs in Los Angeles County in 2035 assuming 100% ZEV for light-duty vehicle.

	2035 Clean	
**Variable**	DAC (N=1173)	non-DAC (N = 1167)
	**Share (%)**	
**Number of Households**	45%	55%
**ZEV Ownership**	46%	54%
**eVMT**	47%	53%
**Pollutant emission reduction**	**(tons/year)**	
**$PM_{2.5}$**	26	29
**NO_x**	134	150
**CO_2**	1,200,000	1,340,000
	**Geometric Mean (IQR)**	
**Traffic-emitted $PM_{2.5}$ concentration ($\mu g/m^3$)[a]**		
**without ZEVs**	0.39 (0.21-0.72)	0.18 (0.095-0.45)
**with ZEVs[b]**	0.29 (0.15-0.54)	0.13 (0.068-0.32)
**reduction attributable to ZEVs**	0.10 (0.053-0.18)	0.049 (0.025 -0.13)
**Traffic-emitted NO_x concentration (ppb)**		
**without ZEVs**	1.6 (0.87-2.9)	0.72 (0.37-1.8)
**with ZEVs[b]**	0.12 (0.060-0.23)	0.05 (0.024-0.13)
**reduction attributable to ZEVs**	1.5 (0.79-2.7)	0.66 (0.34-1.7)

The upper part of the table reports the shares of the number of households, ZEV ownership, simulated eVMT, and corresponding aggregated emission reductions for $PM_{2.5}$, NO_x, and CO_2 in tons per year for 2020 and 2035. The lower part of the table reports model-simulated pollutant concentrations attributable to traffic for $PM_{2.5}$ and NO_x and the reduction attributable to ZEVs in Los Angeles County SB535 DACs and non-DACs.

[a]Average annual daily concentration

[b]ZEVs accounted for 100% of the total light-duty vehicle fleet, 16% medium-duty vehicle, and 20% heavy-duty vehicle in 2035

Table S2 | MATSIM and EMFAC Vehicle Category

MATSIM Vehicle Type	Passenger Vehicles	Light-heavy Truck	Medium-heavy Truck	Heavy-heavy Truck
Corresponding Weight Class	< 8,500 lbs GVW	8,500 to 14,000 lbs. GVW	14,001 to 33,000 lbs. GVW	>33,000 lbs. GVW
EMFAC Vehicle Category	"LDA", "LDT1", "LDT2", "MDV"	"LHD1", "LHD2"	"T6 CAIRP Class 4", "T6 CAIRP Class 5", "T6 CAIRP Class 6", "T6 CAIRP Class 7", "T6 Instate Delivery Class 4", "T6 Instate Delivery Class 5", "T6 Instate Delivery Class 6", "T6 Instate Delivery Class 7", "T6 Instate Other Class 4", "T6 Instate Other Class 5", "T6 Instate Other Class 6", "T6 Instate Other Class 7", "T6 Instate Tractor Class 6", "T6 Instate Tractor Class 7", "T6 OOS Class 4", "T6 OOS Class 5", "T6 OOS Class 6", "T6 OOS Class 7", "T6 Public Class 4", "T6 Public Class 5", "T6 Public Class 6", "T6 Public Class 7", "T6 Utility Class 5", "T6 Utility Class 6", "T6 Utility Class 7", "T6TS"	"T7 CAIRP Class 8", "T7 NNOOS Class 8", "T7 NOOS Class 8", "T7 POAK Class 8", "T7 POLA Class 8", "T7 Public Class 8", "T7 Single Concrete/Transit Mix Class 8", "T7 Single Dump Class 8", "T7 Single Other Class 8", "T7 SWCV Class 8", "T7 Tractor Class 8", "T7 Utility Class 8", "T7IS"
