# Supplementary material for: California’s zero-emission vehicle adoption brings air quality benefits yet equity gaps persist
Source: Nat Commun. 2023 Dec 12;14:7798. doi: 10.1038/s41467-023-43309-9 (PMC10716132; doi:10.1038/s41467-023-43309-9)
Supplement: Supplementary file 3 — Reporting Summary [file 41467_2023_43309_MOESM3_ESM.pdf]

nature portfolio

Corresponding author(s): Yifang Zhu

Last updated by author(s): Oct 24, 2023

Reporting Summary

Nature Portfolio wishes to improve the reproducibility of the work that we publish. This form provides structure for consistency and transparency in reporting. For further information on Nature Portfolio policies, see our Editorial Policies and the Editorial Policy Checklist.

Statistics

For all statistical analyses, confirm that the following items are present in the figure legend, table legend, main text, or Methods section.

n/a	Confirmed	
☐	☒	The exact sample size (*n*) for each experimental group/condition, given as a discrete number and unit of measurement
☒	☐	A statement on whether measurements were taken from distinct samples or whether the same sample was measured repeatedly
☐	☒	The statistical test(s) used AND whether they are one- or two-sided<br>*Only common tests should be described solely by name; describe more complex techniques in the Methods section.*
☒	☐	A description of all covariates tested
☒	☐	A description of any assumptions or corrections, such as tests of normality and adjustment for multiple comparisons
☐	☒	A full description of the statistical parameters including central tendency (e.g. means) or other basic estimates (e.g. regression coefficient) AND variation (e.g. standard deviation) or associated estimates of uncertainty (e.g. confidence intervals)
☐	☒	For null hypothesis testing, the test statistic (e.g. *F*, *t*, *r*) with confidence intervals, effect sizes, degrees of freedom and *P* value noted<br>*Give P values as exact values whenever suitable.*
☒	☐	For Bayesian analysis, information on the choice of priors and Markov chain Monte Carlo settings
☒	☐	For hierarchical and complex designs, identification of the appropriate level for tests and full reporting of outcomes
☐	☒	Estimates of effect sizes (e.g. Cohen's *d*, Pearson's *r*), indicating how they were calculated

Our web collection on statistics for biologists contains articles on many of the points above.

Software and code

Policy information about availability of computer code

Data collection	CalEnviroScreen 4.0: a screening and mapping tool that can be used to help identify California communities that are disproportionately burdened by multiple sources of pollution, developed by California Environmental Protection Agency.<br>EMission FACtor (EMFAC) v1.0.2: a public querying database for California's emissions inventories of onroad and offroad mobile sources and fleet database, developed by California Air Resources Board.<br>Activity Based Model 2016: a new generation of travel demand model released by Southern California Association of Governments.
Data analysis	R-LINE v1.2: a line-source dispersion model developed by the U.S. Environmental Protection Agency using steady-state Gaussian formulation.<br>MATSIM v13: an open-source framework for implementing large-scale agent-based transport simulation.<br>AERMET v22112: an open-source meteorological data preprocessor developed by the U.S. Environmental Protection Agency.<br>Python 3.9.13: an open-source versatile high-level programming language used in web development, data analysis, and AI.<br>Pandas 1.5.2: a Python package for flexible data manipulation and analysis.<br>NumPy 1.23.5: a Python package supporting large, multi-dimensional arrays and matrices.<br>SciPy 1.8.1: a Python package for scientific computing and technical computing.<br>GeoPandas 0.13.2: a Python package for easier handling of geospatial data.<br>QGIS 3.26.2: an open-source software for geospatial data handling and visualization.

For manuscripts utilizing custom algorithms or software that are central to the research but not yet described in published literature, software must be made available to editors and reviewers. We strongly encourage code deposition in a community repository (e.g. GitHub). See the Nature Portfolio guidelines for submitting code & software for further information.

Data

Policy information about availability of data

All manuscripts must include a data availability statement. This statement should provide the following information, where applicable:

- Accession codes, unique identifiers, or web links for publicly available datasets
- A description of any restrictions on data availability
- For clinical datasets or third party data, please ensure that the statement adheres to our policy

The datasets analyzed in this study are sourced from publicly available databases as cited within the manuscript. These resources are open-access and can be freely accessed for further research and validation. Specifically, the Environmental Justice Index and DAC designation can be accessed from California government websites (https://oehha.ca.gov/calenviroscreen/report/calenviroscreen-40 and https://oehha.ca.gov/calenviroscreen/sb535). California ZEV registration data and emission rate data can be found in the EMFAC Database (https://arb.ca.gov/emfac/fleet-db and https://arb.ca.gov/emfac/). Meteorological data are available from the National Oceanic and Atmospheric Administration (https://www.ncei.noaa.gov/data/global-hourly/archive/isd/ and https://rucsoundings.noaa.gov/). The R-LINE modeled hourly traffic-attributable NOx and PM2.5 concentrations, both with and without ZEVs for all scenarios, can be downloaded from https://figshare.com/s/a95749be8bb3bb8700a9.

Research involving human participants, their data, or biological material

Policy information about studies with human participants or human data. See also policy information about sex, gender (identity/presentation), and sexual orientation and race, ethnicity and racism.

Reporting on sex and gender	Not applicable.
Reporting on race, ethnicity, or other socially relevant groupings	Not applicable.
Population characteristics	Not applicable.
Recruitment	Not applicable.
Ethics oversight	Not applicable.

Note that full information on the approval of the study protocol must also be provided in the manuscript.

Field-specific reporting

Please select the one below that is the best fit for your research. If you are not sure, read the appropriate sections before making your selection.

☐ Life sciences ☐ Behavioural & social sciences ☒ Ecological, evolutionary & environmental sciences

For a reference copy of the document with all sections, see nature.com/documents/nr-reporting-summary-flat.pdf

Ecological, evolutionary & environmental sciences study design

All studies must disclose on these points even when the disclosure is negative.

Study description	Our study quantify zero-emission vehicle adoption at the census tract level in California from 2015 to 2020 and project it to 2035 when all passenger vehicles sold are expected to be ZEVs. We then apply an integrated traffic model together with a dispersion model to simulate air quality changes near roads in the Greater Los Angeles area. This study is a modeling exploration that does not involve any laboratory experimentation or field data gathering. The data and models leveraged in this research are derived entirely from publicly accessible databases, and the study employs open-source tools exclusively.
Research sample	The 10% population sample was selected from the synthetic population generated by the Southern California Association of Governments (SCAG). The synthetic population is an artificial group of individuals that represents the population in six counties of Southern California (Los Angeles, Orange, San Bernardino, Ventura, Riverside, and Imperial), which guarantees the representative distributions of demographic and socio-economic characteristics of the real population. Such a population synthesis approach bypasses the challenges of collecting data for the entire population and the potential privacy issue. In this manuscript, we selected 10% of the synthetic population of Los Angeles County based on the residential locations of their households. The 10% population includes both male and female from 0 – 65 years old. There is no organisms involved in the study.
Sampling strategy	The sample of 10% population was selected based on the residential locations of households. Based on the Traffic Analysis Zone (TAZ) system in Los Angeles County, we randomly selected 10% of the households from each TAZ as our sample. The synthetic demographic and socio-economic attributes, as well as the travel information of each household member was selected accordingly and adopted as the input to the transportation model.
Data collection	The data sources include 2010 American Community Survey (ACS), California Department of Finance (DOF), California Employment

Data collection	Development Department (EDD), firm-based InfoGroup data, 2016 Land Use data and County Assessor's Parcel Database. The data was collected by different public agencies and processed by Southern California Association of Governments (SCAG) to generate the synthetic population. We select the research sample from the SCAG's synthetic population of Southern California.
Timing and spatial scale	We did not conduct primary data collection. Our data were drawn from the Southern California Association of Governments (SCAG) synthetic population, based on data from various public agencies with a base year of 2016. We collect the data directly from SCAG which include six counties of Southern California (Los Angeles, Orange, San Bernardino, Ventura, Riverside, and Imperial) in year 2022.
Data exclusions	No data were excluded from analysis.
Reproducibility	The study ensures high reproducibility due to its pure numerical modeling approach, employing publicly accessible raw data and open-source tools. We have applied this methodology for several other projects in the past. The same approach has been replicated in two projects within our lab in 2023, each comprising five scenarios, for a total of 10 instances.
Randomization	Since no sample or participants were allocated in this study, randomization and covariates control are not applicable to this study.
Blinding	No blinding is used in this study. In numerical or computational modeling work, blinding is not necessary because the work does not involve human judgment or interpretation during the data collection or analysis process that could be biased.

Did the study involve field work? ☐ Yes ☒ No

Reporting for specific materials, systems and methods

We require information from authors about some types of materials, experimental systems and methods used in many studies. Here, indicate whether each material, system or method listed is relevant to your study. If you are not sure if a list item applies to your research, read the appropriate section before selecting a response.

Materials & experimental systems

n/a	Involved in the study	
☒	☐	Antibodies
☒	☐	Eukaryotic cell lines
☒	☐	Palaeontology and archaeology
☒	☐	Animals and other organisms
☒	☐	Clinical data
☒	☐	Dual use research of concern
☒	☐	Plants

Methods

n/a	Involved in the study	
☒	☐	ChIP-seq
☒	☐	Flow cytometry
☒	☐	MRI-based neuroimaging
